# Comparison of Body Composition, Basal Metabolic Rate and Metabolic Outcomes of Adults with Prader-Willi Syndrome and Age- and BMI-Matched Patients with Essential Obesity

**DOI:** 10.3390/jcm14082646

**Published:** 2025-04-12

**Authors:** Stefano Lazzer, Alessandro Gatti, Mattia D’Alleva, Lara Mari, Simone Zaccaron, Jacopo Stafuzza, Enrico Rejc, Adele Bondesan, Diana Caroli, Francesca Frigerio, Laura Abbruzzese, Enrica Ventura, Graziano Grugni, Alessandro Sartorio

**Affiliations:** 1 Department of Medicine, University of Udine, 33100 Udine, Italy; mattia.dalleva@uniud.it (M.D.); mari.lara@spes.uniud.it (L.M.); simone.zaccaron@uniud.it (S.Z.); jacopo.stafuzza@uniud.it (J.S.); enrico.rejc@uniud.it (E.R.); 2 School of Sport Sciences, University of Udine, 33100 Udine, Italy; 3 Laboratory of Adapted Motor Activity (LAMA), Department of Public Health, Experimental and Forensic Medicine, University of Pavia, 27100 Pavia, Italy; alessandro.gatti08@universitadipavia.it; 4 National PhD Programme in One Health Approaches to Infectious Diseases and Life Science Research, Department of Public Health, Experimental and Forensic Medicine, University of Pavia, 27100 Pavia, Italy; 5 Department of Neurosciences, Biomedicine and Movement Sciences, University of Verona, 37124 Verona, Italy; 6 Experimental Laboratory for Auxo-Endocrinological Research, Istituto Auxologico Italiano, Istituto di Ricovero e Cura a Carattere Scientifico (IRCCS), 28824 Piancavallo-Verbania, Italy; a.bondesan@auxologico.it (A.B.); d.caroli@auxologico.it (D.C.); f.frigerio@auxologico.it (F.F.); g.grugni@auxologico.it (G.G.); sartorio@auxologico.it (A.S.); 7 Division of Auxology, Istituto Auxologico Italiano, Istituto di Ricovero e Cura a Carattere Scientifico (IRCCS), 28824 Piancavallo-Verbania, Italy; l.abbruzzese@auxologico.it; 8 Division of Eating and Nutrition Disorders, Istituto Auxologico Italiano, Istituto di Ricovero e Cura a Carattere Scientifico (IRCCS), 28824 Piancavallo-Verbania, Italy; e.ventura@auxologico.it

**Keywords:** obesity, metabolic syndrome, Prader-Willi syndrome, body composition, basal metabolic rate

## Abstract

**Background/Objectives**: This study compared metabolic syndrome (MetS) features in patients with Prader-Willi syndrome (PWS) to those in age-, BMI-, and gender-matched subjects with essential obesity (EOB). **Methods**: Thirty-two PWS patients (23 females, 9 males; median age 31.6 years; BMI 42.0 kg/m^2^) underwent several assessments, including anthropometric measurements, body composition via bio-impedance analysis, basal metabolic rate (BMR) using indirect calorimetry, and blood sampling. **Results**: Their data were compared to a matched EOB group (23 females, 9 males; median age 31.4 years; BMI 43.5 kg/m^2^). The study groups (PWS and EOB) were subsequently divided into two subgroups based on the International Diabetes Federation criteria for the definition of MetS. Results showed that individuals with PWS had significantly lower (*p* < 0.001) body weight (BW, −20.9%), height (−8.9%), fat-free mass (FFM, −23.5%), and fat mass (FM, −19.2%) in absolute terms compared to EOB subjects. However, the relative percentages of FFM and FM were similar. Absolute BMR was 25.5% (*p* < 0.001) lower in the PWS group; however, this difference disappeared when adjusted for FFM or body weight (BW). Metabolic outcomes were broadly similar between the groups, except for higher fasting glucose (+7.3%) and HbA1c levels (+7.9%), and lower fasting insulin (−29.0%) in PWS patients. **Conclusions**: Moreover, PWS subjects exhibited higher total cholesterol (+9.6%) and HDL-cholesterol (+19.8%), suggesting a more favourable lipid profile and no extra risk beyond severe obesity.

## 1. Introduction

Prader-Willi Syndrome (PWS) is a rare genetic disorder characterised by a complex array of clinical features, including hypotonia, developmental delay, behavioral abnormalities, multiple endocrinopathies (hypothyroidism, growth hormone (GH) deficiency, hypogonadism, central adrenal insufficiency), hyperphagia, and obesity [1]. This syndrome results from the loss of expression of paternal genes on the PWS critical region on chromosome 15 (15q11-13), typically due to a paternal deletion, maternal uniparental disomy, or imprinting defects [2]. One of the primary consequences of PWS is obesity, which is particularly distinctive because it occurs early in childhood, is relentless and leads to severe morbidities if left unchecked [3]. As a result, patients with PWS are at a heightened risk for various metabolic complications, including insulin resistance, dyslipidemia, and hypertension, which collectively form a condition known as Metabolic Syndrome (MetS) [4]. Furthermore, cardiovascular comorbidities are frequently observed in subjects with PWS, including heart failure, coronary heart disease and atrial fibrillation [5].

MetS is a cluster of cardiovascular risk factors that significantly increase the likelihood of developing cardiovascular disease, type 2 diabetes, and stroke. It is characterised by the presence of abdominal obesity, hypertension, elevated fasting glucose levels, high triglyceride levels, and low HDL cholesterol levels [6]. While MetS is prevalent in the general population, its association with genetic conditions, such as PWS, is not well understood, and research on the subject remains relatively sparse.

Essential obesity (EOB), on the other hand, refers to a form of obesity that is not linked to any specific underlying genetic or endocrine disorder, and it is the most common form of obesity in the general population [7]. The pathogenesis of EOB is multifactorial, involving interactions between genetic susceptibility, environmental factors, and lifestyle choices. However, obesity in patients with EOB typically develops due to excessive caloric intake, sedentary behaviour and genetic predispositions [7,8], rather than the specific endocrine dysfunctions observed in genetic syndromes like PWS.

The metabolic challenges faced by patients with PWS differ from those seen in patients with EOB due to the underlying genetic and endocrine abnormalities present in PWS. The early onset and progressive nature of obesity in PWS are thought to be driven, in part, by hypothalamic dysfunction, which disrupts normal satiety signals and leads to an uncontrollable drive to eat. In contrast, patients with EOB may have an altered balance of appetite-regulating hormones; however, the root causes are more closely linked to external factors, such as diet and physical activity. The combination of hyperphagia, reduced physical activity, and endocrine dysfunction in PWS creates a unique metabolic profile that differs substantially from the more common metabolic disturbances observed in EOB [9].

This article compares the MetS features in patients with PWS and those with EOB. This comparison is essential not only to refine clinical care for patients with PWS but also to provide insights into a broader understanding of the complex interactions between genetics, metabolism, and obesity.

## 2. Materials and Methods

### 2.1. Patients

The study is retrospective. The period of recruitment of the study population was between September 2021 and October 2022. Patients were selected from the groups of patients hospitalized at the Division of Auxology (patients with PWS) and the Division of Metabolic Disorders (patients with EOB) at the Istituto Auxologico Italiano in Piancavallo-Verbania, Italy, for a 3-week multidisciplinary body weight reduction program. Initially, patients diagnosed with PWS were chosen; afterwards, a control group was formed by selecting patients with EOB, matched by age and body mass index (BMI).

Patients with PWS exhibited the characteristic clinical traits of the syndrome, with their diagnosis confirmed through cytogenetic evaluation (interstitial deletion of the paternal chromosome 15, del15q11.2-q13 (DEL15) = 20 cases; maternal uniparental disomy of chromosome 15 (UPD15) = 12 cases). The eligibility criteria included: (1) both genders; (2) age > 18 years; (3) BMI > 30 kg/m^2^ (for both groups, PWS and EOB). Exclusion criteria were: (1) obesity due to secondary causes (e.g., steroid-induced obesity, untreated hypothyroidism, etc.), except for PWS; (2) any major cardiovascular, psychiatric, neurological, or other serious medical conditions detected in the previous six months; (3) refusal to sign the informed consent form by the patients and by the parents of patients with PWS. In addition, concerning the group of PWS, seven subjects were undergoing GH therapy (MetS+/MetS−: 3/4), 14 were receiving sex steroid replacement therapy (MetS+/MetS−: 8/6), and four were treated with levo-thyroxine for hypothyroidism (MetS+/MetS−: 1/3). Only one patient with EOB was treated with levo-thyroxine. All the subjects performed a low level of physical activity (1–2 h/week) in the three months before their admission to our hospital. No significant changes in body weight were observed during the 3 months preceding hospitalization.

Anthropometric assessments, basal metabolic rate assessment, body composition analysis, and blood sample collections were performed in the first 3 days after admission to the Hospital.

The study protocol was reviewed and approved by the Territorial Ethical Committee (CET 5), Lombardy Region, Milan, Italy (EC code: 214/24; research project code: 01C410; acronym: REEPWSOB).

### 2.2. Basal Metabolic Rate

Basal metabolic rate (BMR) was assessed following an overnight fast using an open-circuit, indirect computerized calorimetry system (Vmax 29, Sensor Medics, Yorba Linda, CA, USA) equipped with a rigid, transparent, and ventilated canopy. The medical charts of fertile women were reviewed for regularity of menses and the date of the last menstrual period. BMR was always determined during the follicular phase of the menstrual cycle. Energy expenditure was calculated from O_2_ oxygen uptake and CO_2_ output using the equation of Weir [10].

### 2.3. Anthropometric Assessments

Weight was recorded using a standard scale, ensuring a precision of 0.1 kg. Standing height was measured using a Harpenden Stadiometer (Holtain Limited, Crymych, Dyfed, UK). Body mass index (BMI) was determined as weight (kg) divided by height squared (m^2^). Waist circumference (WC) was measured using a flexible measuring tape while the subject stood upright, positioning the tape midway between the lower edge of the ribs and the upper boundary of the iliac crest.

### 2.4. Body Composition Analysis

Body composition was evaluated through bioelectrical impedance analysis (BIA, Human-IM Scan, DS-Medigroup, Milan, Italy) according to the method of Lukaski et al. [11] after 20 min of rest in a supine position with relaxed arms and legs. FFM and FM were estimated using the prediction equation developed by Gray et al. [12] for male patients and by Bedogni et al. [13] for female patients.

### 2.5. Blood Sample Collection

Blood samples were collected from participants following a standardized procedure at the start of the weight loss program. Samples were drawn into lithium heparin tubes at approximately 8:00 AM after an overnight fast. Plasma was separated from blood cells via centrifugation (20–24 °C, 10 min at 2500× *g*) within two hours of collection. The following biomarkers were measured: total cholesterol (Total-C), high-density lipoprotein cholesterol (HDL-C), low-density lipoprotein cholesterol (LDL-C), triglycerides (TG), glucose, insulin, and C-reactive protein (CRP). Serum Total-C, LDL-C, HDL-C, and TG levels were assessed using colorimetric enzymatic assays (Roche Diagnostics, Monza, Italy), with sensitivities of 3.86 mg/dL [1 mg/dL = 0.03 mmol/L], 3.87 mg/dL [1 mg/dL = 0.03 mmol/L], 3.09 mg/dL [1 mg/dL = 0.03 mmol/L], and 8.85 mg/dL [1 mg/dL = 0.01 mmol/L], respectively. Serum glucose concentration was determined using the glucose oxidase enzymatic method (Roche Diagnostics, Monza, Italy), with a sensitivity of 2 mg/dL [1 mg/dL = 0.06 mmol/L]. Insulin levels were quantified using a chemiluminescent immunometric assay with a commercial kit (Elecsys Insulin, Roche Diagnostics, Monza, Italy), which had a sensitivity of 0.2 μU/mL [1 μU/mL = 7.18 pmol/L]. The intra- and inter-assay coefficients of variation (CVs) were as follows: T-C: 1.1% and 1.6%, LDL-C: 1.2% and 2.5%, HDL-C: 1.8% and 2.2%, TG: 1.1% and 2.0%, Glucose: 1.0% and 1.3%, Insulin: 1.5% and 4.9%. CRP levels were measured via an immunoturbidimetric assay (CRPRX, Roche Diagnostics GmbH, Mannheim, Germany) with a sensitivity of 0.03 mg/dL. Additionally, for each participant, insulin resistance was evaluated using the homeostatic model assessment (HOMA-IR), calculated using the following equation: insulin [μU/mL] × glucose [mmol/L]/22.5.

### 2.6. Blood Pressure Assessment

Blood pressure readings were taken from the right arm using a sphygmomanometer with an appropriately sized cuff while the subject was seated and relaxed. Measurements were conducted three times at 10-min intervals, and the average of the three readings was recorded for both systolic (SBP) and diastolic blood pressure (DBP).

### 2.7. Definition of Metabolic Syndrome and Cardiometabolic Risk Z-Score

According to the literature [6], participants were classified as having MetS if they had three or more of the following altered factors:

i. Abdominal obesity (WC ≥ 102 cm for males; ≥88 cm for females);
ii. Elevated triglycerides: ≥150 mg/dL (1.7 mmol/L) or specific treatment for this lipid abnormality;
iii. Reduced HDL-C: <40 mg/dL (1.0 mmol/L) in males; <50 mg/dL (1.3 mmol/L) in females, or specific treatment for this lipid abnormality;
iv. Increased BP: SBP ≥ 130 mmHg or DBP ≥ 85 mmHg and/or the treatment of previously diagnosed hypertension;
v. Increased fasting plasma glucose (FPG) concentration of ≥100 mg/dL (5.6 mmol/L) or previously diagnosed type 2 diabetes mellitus.

The cardiometabolic risk score reflects the pathophysiologic processes of the Metabolic Syndrome (MetS), particularly in relation to cardiovascular and type 2 diabetes risk [14]. The equation to calculate the MetS z-score requires HDL cholesterol, fasting triglycerides, fasting glucose, BMI z-score, and systolic blood pressure, and it was calculated as follows:

*For men*: MetS z-score = [(40 − HDL-C)/SD] + [(TG − 150)/SD] + [(glucose − 100)/SD] + [(WC − 94)/SD] + [(SBP − 130)/SD] + [(DBP − 85)/SD]

*For women*: MetS z-score = [(50 − HDL-C)/SD] + [(TG − 150)/SD] + [(glucose − 100)/SD] + [(WC − 80)/SD] + [(SBP − 130)/SD] + [(DBP − 85)/SD]

### 2.8. Visceral Adiposity Index

The visceral adiposity index (VAI) is an innovative index that estimates visceral adiposity in adults with obesity, which is strongly associated with various cardiometabolic factors [15]. The equation to compute it is presented below and requires waist circumference, BMI, triglycerides, and HDL cholesterol:

For men: VAI = (WC/(39.68 + (1.88 × BMI))) × (TG/1.03) × (1.31/HDL)

For women: VAI = (WC/(36.58 + (1.89 × BMI))) × (TG/0.81) × (1.52/HDL)

### 2.9. Statistical Analysis

Data were expressed as mean (95% confidence interval, CI). The Shapiro-Wilk test was used to evaluate the normality of the data. Sphericity was verified by Mauchly’s test. A Greenhouse-Geisser correction was applied in cases where the sphericity assumption was violated. Comparisons between the PWS and EOB groups were conducted using parametric or non-parametric analysis of covariance (ANCOVA), adjusting for age and sex. For the multiple groups’ comparisons (PWS with or without MetS vs. EOB with or without MetS), the same ANCOVA model was applied with a post-hoc analysis using Tukey or Bonferroni depending on the data distribution. For all analyses, a *p*-value < 0.05 was considered statistically significant. Analyses were conducted using R software (version 4.4, R Foundation for Statistical Computing). The “Durga” package was used to generate the group comparison plots.

## 3. Results

### 3.1. Comparison Between Patients with PWS and Patients with EOB

The two groups were matched for age, BMI and gender. The prevalence of women was higher in both groups; therefore, all analyses were adjusted for gender.

PWS patients showed lower body weight (BW, −20.9%, *p* < 0.001), height (−8.9%, *p* < 0.001), absolute FFM (−23.5%, *p* < 0.001) and FM (−19.2%, *p* < 0.001) than in patients with EOB, whereas relative (%) FFM and FM were not significantly different between the two groups (Table 1). Absolute BMR was significantly lower in patients with PWS (−25.5%, *p* < 0.001) than in patients with EOB; the difference between the two groups disappeared when BMR was adjusted for FFM or BW (Table 1).

As reported in Table 2, patients with PWS showed significantly higher fasting glucose (+7.3%, *p*: 0.017) and Hb1Ac levels (+7.9%, *p* < 0.001), and lower fasting insulin (−29.0%, *p* < 0.002), while the HOMA-IR index was comparable in the two groups (Table 2). As far as the lipid profile was concerned, patients with PWS showed significantly higher total cholesterol (+9.6%, *p*: 0.049) and HDL-cholesterol (+19.8%, *p* < 0.001), while LDL-cholesterol and triglyceride levels were similar in the two groups (Table 2). No significant differences were observed between the two groups in C-reactive protein, waist circumferences, systolic and diastolic blood pressure, cardiometabolic risk score, or visceral adiposity index (Table 2).

The study groups (PWS and EOB) were divided into two subgroups based on the IDF criteria for the definition of MetS: those who met the criteria (PWS MetS+, n. 16, 50%; EOB MetS+, n. 16, 50%) and those who did not (PWS MetS−, n. 16, 50%; EOB MetS−, n. 16, 50%). Six out of 32 subjects with PWS (all in the PWS MetS+ subgroup) and 4 subjects with EOB (all in the EOB MetS+ subgroup) suffered from T2DM and were treated with oral hypoglycemic drugs. In the whole PWS group, the most frequently altered parameters were WC (32 out of 32 patients, 100%), followed by BP (14/32, 44%) and HDL-C (11/32, 34%), while TG was altered in 9/32 patients (28%) and fasting glucose (FG) in 8/32 patients (25%). In patients with PWS (Mets+), the most frequently altered parameters were WC (16 out of 16 patients, 100%), followed by HDL-C (12/16, 75%) and TG (11/16, 69%), while FG and BP were altered in 10/16 patients (63%), respectively. In the whole EOB group, the most frequently altered parameters were WC (32 out of 32 patients, 100%), followed by HDL-C (22/32, 69%) and BP (19/32, 59%), while TG was altered in 10/32 patients (31%) and FG in 4/32 patients (13%). In patients with EOB (Mets+), the most frequently altered parameters were WC (16 out of 16 patients, 100%), followed by HDL-C (15/16, 94%) and BP (13/16, 81%), while TG was altered in 9/16 patients (56%) and FG in 4/16 patients (25%).

### 3.2. Comparison Between Patients with PWS and Patients with EOB, with Metabolic Syndrome (MetS+) or Without Metabolic Syndrome (MetS−)

Individuals with PWS (MetS+) exhibited lower BW (−20.4%, *p* < 0.05), height (−9.4%, *p* < 0.05), absolute FFM (−26.2%, *p* < 0.05) and BMR (−23.5%, *p* < 0.05) than individuals with EOB (MetS+). In contrast, relative FFM, FM and BMR adjusted for FFM or BW were not significantly different between the two groups (Table 3).

Individuals with PWS (MetS−) showed lower BW (−20.9%, *p* < 0.05), height (−9.0%, *p* < 0.05); absolute FFM (−19.5%, *p* < 0.05) and FM (−23.0%, *p* < 0.05), and BMR (−27.2%, *p* < 0.05) than individuals with EOB (MetS−). By contrast, relative FFM and FM, as well as BMR adjusted for FFM or BW, were not significantly different between the two groups (Table 3).

Individuals with PWS (MetS+) and PWS (MetS−), as well as individuals with EOB (MetS+) and EOB (MetS−), exhibited similar anthropometric characteristics, body composition, and BMR (Table 3).

Subjects with PWS (MetS+) showed lower fasting insulin (−27.9%, *p* < 0.05), higher fasting glucose (+12.1%, *p* < 0.05), Hb1Ac (+12.1%, *p* < 0.05) and the same HOMA-IR than subjects with EOB (MetS+) (Table 4). No other significant differences were observed between individuals with PWS (MetS+) and those with EOB (MetS+).

Individuals with PWS (MetS−) showed higher HDL-cholesterol (+18.9%, *p* < 0.05) than those with EOB (MetS−). No other significant differences were observed between the two subgroups (Table 4).

Individuals with PWS (MetS+) showed higher fasting glucose (+11.8%, *p* < 0.05), Hb1Ac (+10.2%, *p* < 0.05), HOMA-IR (+44.8%, *p* < 0.05) and triglycerides (+44.1%, *p* < 0.05) levels, and lower HDL-cholesterol (−20.1%, *p* < 0.05) than individuals with PWS (MetS−), while no significant differences were recorded in fasting insulin or total- and LDL-cholesterol levels between the two subgroups (Table 4). Individuals with PWS (MetS+) exhibited a higher systolic blood pressure (+8.4%, *p* < 0.05) and visceral adiposity index (−58.6%, *p* < 0.05) than individuals with PWS (MetS−) (Table 4). Individuals with EOB (MetS+) and those with EOB (MetS−) showed the same metabolic outcomes (Table 4).

## 4. Discussion

In the present study, we compared body composition, BMR and metabolic outcomes in individuals with PWS and in subjects with EOB, with or without MetS.

Individuals with PWS exhibited significantly lower BW and stature compared to age-, gender- and BMI-matched subjects with EOB, as previously observed [16]. Furthermore, individuals with PWS had lower absolute values of both FFM and FM than those recorded in subjects with EOB, while the relative FFM and FM values were comparable between the two groups. This last finding was in contrast with previous studies [17,18,19] demonstrating that differences in body composition between individuals with PWS and subjects with EOB could be related to the different characteristics of the study groups in terms of BMI, age, gender and hormonal replacement therapies (for individuals with PWS). BMR was lower in absolute terms in individuals with PWS than in subjects with EOB, but it was comparable when adjusted for FFM or BW, thus suggesting a lack of specific syndromic-related effects on BMR. Furthermore, the close relationship between BMR and FFM suggests a major role of FFM as a determinant of BMR, as previously described [20,21].

The metabolic outcomes of subjects with PWS were not significantly different than those recorded in individuals with EOB, except for lower fasting insulin levels and elevated fasting glucose and HbA1c levels than in individuals with EOB. This apparent paradox could reflect a particular insulin sensitivity in subjects with PWS, which could be related to a possible alteration in the adipocyte profile, with higher levels of adiponectin contributing to greater insulin sensitivity [22]. Moreover, individuals with PWS are inclined to accumulate less visceral fat compared to those with EOB, and visceral fat is closely associated with insulin resistance [16]. This finding is consistent with the reduced insulinemia observed in subjects with PWS, and may reflect a balance between factors that promote and counteract insulin resistance in this population [23]. The higher levels of HDL cholesterol in individuals with PWS than in subjects with EOB suggest a potentially more favourable and cardioprotective lipid profile. The higher total cholesterol is probably due to the increase in the HDL fraction rather than a greater LDL presence. This characteristic could be influenced by the different distribution of fat in individuals with PWS, who have a greater accumulation of subcutaneous fat and a lower amount of visceral fat, supporting a more favourable lipid metabolism, in agreement with previous reports [23].

In the present study, subjects with PWS (MetS+) exhibited lower BW and height, along with lower absolute values of both FFM. In contrast, the relative values of FFM and FM remained comparable to subjects with EOB (MetS+). Absolute BMR was lower in individuals with PWS (MetS+) than in subjects with EOB (MetS+); however, when adjusted for FFM or BW, BMR was comparable between the two groups, showing no specific impairments in individuals with PWS [20,21]. Conversely, metabolic assessments showed lower fasting insulin levels and higher fasting glucose and HbA1c levels in individuals with PWS (MetS+) than in subjects with EOB (MetS+), with no significant differences in HOMA-IR, suggesting higher insulin sensitivity in individuals with PWS (MetS+) [24].

Individuals with PWS (MetS−) exhibited lower BW and height, and had reduced absolute fat-free mass (FFM) and fat mass (FM) compared to subjects with EOB (MetS−), while the relative percentages of FFM and FM remained similar between the two subgroups. Absolute BMR was lower in individuals with PWS (MetS−) than in subjects with EOB (MetS−). However, when normalized for FFM or BW, it was comparable between the two subgroups as observed previously [20,21]. Metabolic and lipid profiles; including fasting insulin, fasting glucose, HbA1c, and HOMA-IR; total cholesterol, LDL-cholesterol, and triglyceride levels were comparable between individuals with PWS (MetS−) and subjects with EOB (MetS−), with the exception of HDL-cholesterol levels, which were higher in individuals with PWS (MetS−) than in subjects with EOB (MetS−), thus suggesting a better protection factor from cardiovascular diseases, such as ischemic stroke and myocardial infarction, in individuals with PWS (MetS−) [24].

Finally, individuals with PWS (MetS+) had similar anthropometric characteristics, body composition and BMR to individuals with PWS (MetS−), suggesting that these parameters are not able to predict the development of MetS as shown previously [25]. As expected, individuals with PWS (MetS+) exhibited higher HbA1c and HOMA-IR values compared to individuals with PWS (MetS−), underlining their increased risk for developing type 2 diabetes and other metabolic complications [26]. Individuals with PWS (MetS+) showed higher VAI values than subjects with PWS (MetS−), highlighting their increased cardiovascular risk [27].

The strength of this study is that all patients with PWS and with EOB were recruited by a single center, with the same well-trained operators and the same laboratory, which makes data interpretation more reliable than that obtained in a multicenter study. On the other hand, we acknowledge some limitations in our study. Firstly, the number of enrolled subjects is small, thus resulting in limited strength of the statistical analysis. Nevertheless, PWS is a rare disease, and enrollment of large experimental samples is difficult. Another weak point is the lack of evaluation of body composition by DEXA, which represents one of the best available techniques for its evaluation. In this respect, however, it has been previously reported that BIA can be used to estimate body composition in both men and women with PWS, applying population-specific equations [13].

In conclusion, the metabolic differences observed between adults with PWS and those with EOB suggest that: i. The reduced BMR in patients with PWS was primarily due to the lower FFM rather than to a specific metabolic alteration; ii. Despite elevated fasting glucose and HbA1c levels, individuals with PWS exhibit lower insulin levels, indicating a distinct insulin sensitivity, possibly influenced by adiponectin and lower visceral fat; iii. The lipid profile of individuals with PWS appears more favorable than that recorded in individuals with EOB. Moreover, the overall cardiometabolic risk and insulin resistance (HOMA-IR) were similar between the two groups, suggesting that PWS does not pose an additional risk beyond that associated with obesity.

On the other hand, our findings raise the need for a timely therapeutic approach in obese adults with PWS, including personalized physical activity and nutritional intervention, in addition to drug therapy. Considering that their life expectancy has increased substantially in recent years [28], this is necessary in order to avoid a significant increase in the development of obesity-related diseases in the near future.

## Figures and Tables

**Table 1:** Anthropometric characteristics, body composition and basal metabolic rate of individuals with essential obesity (EOB) and individuals with Prader-Willi Syndrome (PWS).

	All (*n* = 64)	EOB (*n* = 32)	PWS (*n* = 32)	*p*-Value
Female (*n* (%))	46 (71.9%)	23 (71.9%)	23 (71.9%)	-
Age (years)	31.5 (29.5, 33.4)	31.3 (29.0, 33.7)	31.6 (28.4, 34.8)	0.901
Body weight (kg)	110.3 (105.4, 115.1)	123.1 (118.8, 127.5)	97.4 (91.5, 103.3)	0.001
Height (cm)	1.60 (1.58, 1.63)	1.68 (1.65, 1.71)	1.53 (1.49, 1.56)	0.001
Body mass index (kg/m^2^)	42.8 (41.4, 44.1)	43.5 (42.3, 44.7)	42.0 (39.6, 44.4)	0.299
Fat-free mass (kg)	52.5 (49.8, 55.2)	59.5 (56.1, 62.9)	45.5 (43.0, 48.0)	0.001
Fat free mass (%)	48.1 (46.6, 49.5)	48.3 (46.4, 50.2)	47.86 (45.7, 49.9)	0.721
Fat mass (kg)	57.6 (54.4, 60.8)	63.7 (60.5, 67.0)	51.50 (46.8, 56.1)	0.001
Fat mass (%)	52.1 (50.7, 53.5)	51.8 (49.9, 53.7)	52.38 (50.3, 54.4)	0.664
Basal metabolic rate (kcal)	1824 (1726, 1923)	2096 (1993, 2199)	1561 (1458, 1664)	0.001
Basal metabolic rate (kcal/kg FFM)	34.9 (33.8, 36.0)	36.0 (34.6, 37.4)	33.8 (32.2, 35.5)	0.054
Basal metabolic rate (kcal/kg BW)	16.58 (16.08, 17.09)	17.03 (16.52, 17.54)	16.15 (15.30, 17.00)	0.080

Data are presented as mean (95% confidence interval). *p*-value was retrieved from a two-sided ANCOVA, adjusting for age and gender.

**Table 2:** Metabolic outcomes of individuals with essential obesity (EOB) and individuals with Prader-Willi Syndrome (PWS).

	All (*n* = 64)	EOB (*n* = 32)	PWS (*n* = 32)	*p*-Value
Fasting glucose (mg/dL)	89.8 (87.0, 92.6)	86.8 (83.5, 90.1)	93.1 (88.8, 97.5)	0.017
Fasting insulin (mU/L)	17.81 (15.76, 19.85)	20.88 (17.78, 23.97)	14.83 (12.53, 17.13)	0.002
Hb1Ac (%)	5.52 (5.39, 5.65)	5.33 (5.22, 5.43)	5.75 (5.52, 5.98)	0.001
HOMA-IR	4.12 (3.64, 4.60)	4.44 (3.80, 5.07)	3.82 (3.11, 4.53)	0.193
Total cholesterol (mg/dL)	172.5 (164.6, 180.4)	164.6 (153.4, 175.8)	180.4 (169.7, 191.1)	0.049
LDL-cholesterol (mg/dL)	110.9 (103.8, 118.0)	104.8 (94.2, 115.4)	117.0 (108.0, 126.1)	0.085
HDL-cholesterol (mg/dL)	44.5 (41.8, 47.1)	40.4 (37.4, 43.4)	48.4 (44.4, 52.3)	0.001
Triglycerides (mg/dL)	122.6 (108.6, 136.6)	117.6 (103.0, 132.2)	127.5 (103.7, 151.2)	0.853
Reactive protein C (mg/dL)	0.96 (0.78, 1.14)	0.81 (0.59, 1.03)	1.12 (0.85, 1.39)	0.093
Waist circumference (cm)	119 (116, 122)	117 (113, 121)	121 (116, 125)	0.173
Systolic blood pressure (mmHg)	127 (124, 129)	128 (124, 131)	126 (121, 130)	0.103
Diastolic blood pressure (mmHg)	81 (80, 83)	83 (80, 86)	80 (78, 81)	0.051
Cardiometabolic risk score	1.45 (0.66, 2.25)	1.77 (0.70, 2.84)	1.12 (−0.07, 2.31)	0.435
Visceral adiposity index	2.49 (2.13, 2.85)	2.43 (2.02, 2.83)	2.55 (1.95, 3.15)	0.696

Data are presented as mean (95% confidence interval). HbA1c = glycated haemoglobin; HOMA-IR = homeostatic model assessment of insulin resistance; LDL-cholesterol = low-density lipoprotein cholesterol; HDL-cholesterol = high-density lipoprotein cholesterol. *p*-value was retrieved from a two-sided ANCOVA, adjusting for age and gender.

**Table 3:** Anthropometric characteristics, body composition and basal metabolic rate of individuals with essential obesity (EOB) and individuals with Prader-Willi Syndrome (PWS), with Metabolic Syndrome (MetS+) or without Metabolic Syndrome (MetS−).

	OB MetS+ (*n* = 16)	PWS MetS+ (*n* = 16)	OB MetS− (*n* = 16)	PWS MetS− (*n* = 16)
Female (*n* (%))	12 (66.7%)	11 (68.8%)	11 (78.6%)	12 (75.0%)
Age (years)	30.9 (27.5, 34.2)	31.7 (27.9, 35.6)	31.9 (28.5, 35.3)	31.50 (26.32, 36.68)
Body weight (kg)	125.2 (120.6, 129.7) ^a,c^	99.6 (90.1, 109.2) ^a,d^	120.5 (112.4, 128.6) ^b,d^	95.24 (88.06, 102.41) ^b,c^
Height (cm)	1.70 (1.66, 1.74) ^a,c^	1.54 (1.50, 1.58) ^a,d^	1.66 (1.62, 1.70) ^b,d^	1.51 (1.47, 1.56) ^b,c^
Body mass index (kg/m^2^)	43.4 (41.9, 45.0)	42.0 (38.4, 45.5)	43.5 (41.5, 45.5)	42.1 (38.7, 45.5)
Fat free mass (kg)	62.1 (57.7, 66.5) ^a,c^	45.8 (42.0, 49.6) ^a,d^	56.2 (51.3, 61.1) ^b,d^	45.2 (41.9, 48.5) ^b,c^
Fat free mass (%)	49.5 (47.0, 52.0)	47.3 (44.0, 50.6)	46.7 (43.8, 49.6)	48.3 (45.6, 51.0)
Fat mass (kg)	63.3 (60.0, 66.6) ^a^	53.4 (45.8, 60.9)	64.3 (58.0, 70.5) ^b^	49.5 (44.1, 54.9) ^a,b^
Fat mass (%)	50.7 (48.2, 53.2)	53.1 (49.9, 56.3)	53.2 (50.3, 56.1)	51.6 (48.9, 54.3)
Basal metabolic rate (kcal)	2172 (2043, 2300) ^a,c^	1662 (1504, 1821) ^a,d^	2003 (1844, 2162) ^b,d^	1458 (1342, 1575) ^b,c^
Basal metabolic rate (kcal/kg FFM)	36.1 (34.1, 38.0)	35.4 (33.2, 3.6)	35.8 (33.7, 37.9)	32.4 (30.2, 34.6)
Basal metabolic rate (kcal/kg BW)	17.39 (16.58, 18.20)	16.87 (15.59, 18.15)	16.60 (16.10, 17.09)	15.43 (14.40, 16.46)

Data are presented as mean (95% confidence interval). *p*-value was retrieved from a two-sided ANCOVA, adjusted for age and gender and with a post-hoc analysis of Tukey or Bonferroni depending on outcome normality. ^a, b, c, d^: Similar letters represent significant differences between groups (*p*  <  0.05).

**Table 4:** Metabolic outcomes in individuals with essential obesity (EOB) and individuals with Prader-Willi Syndrome (PWS), with Metabolic Syndrome (MetS+) or without Metabolic Syndrome (MetS−).

	OB MetS+ (*n* = 16)	PWS MetS+ (*n* = 16)	OB MetS− (*n* = 16)	PWS MetS− (*n* = 16)
Fasting glucose (mg/dL)	87.8 (82.9, 92.7) ^a^	99.9 (92.2, 107.6) ^a,b,c^	85.6 (81.2, 90.0) ^b^	88.1 (84.7, 91.5) ^c^
Fasting insulin (mU/L)	24.10 (20.18, 28.02) ^a,b^	17.36 (14.14, 20.57) ^a^	16.96 (12.75, 21.18)	12.31 (9.44, 15.18) ^b^
Hb1Ac (%)	5.38 (5.23, 5.53) ^a^	6.12 (5.68, 6.56) ^a,b,c^	5.26 (5.11, 5.41) ^b^	5.49 (5.35, 5.64) ^c^
HOMA-IR	5.14 (4.36, 5.93) ^a^	4.93 (3.95, 5.90) ^b^	3.58 (2.72, 4.43)	2.72 (2.01, 3.43) ^a,b^
Total cholesterol (mg/dL)	170.5 (157.0, 184.0)	179.4 (162.8, 196.0	157.0 (138.3, 175.7)	181.4 (167.5, 195.3)
LDL-cholesterol (mg/dL)	111.6 (100.3, 123.0)	117.1 (102.6, 131.5)	96.0 (77.1, 114.8)	117.0 (105.6, 128.4)
HDL-cholesterol (mg/dL)	38.1 (34.4, 41.8) ^a^	43.0 (38.5, 47.5) ^b^	43.6 (38.9, 48.3) ^c^	53.8 (48.3, 59.2) ^a,b,c^
Triglycerides (mg/dL)	133.3 (113.5, 153.1)	163.5 (125.0, 202.1) ^a,b^	98.6 (81.0, 116.2) ^a^	91.4 (77.7, 105.0) ^b^
Reactive protein C (mg/dL)	0.78 (0.45, 1.11)	1.20 (0.88, 1.52)	0.84 (0.55, 1.14)	1.04 (0.62, 1.47)
Waist circumference (cm)	120 (115, 125)	123 (117, 129)	113.79 (108, 119)	119 (112, 125)
Systolic blood pressure (mmHg)	130 (125, 133) ^a^	131 (123, 138) ^b^	125 (121, 128)	120 (118, 123) ^a,b^
Diastolic blood pressure (mmHg)	85 (82, 89)	80 (79, 82)	79 (75, 83)	79 (77, 81)
Cardiometabolic risk score	2.94 (1.50, 4.39) ^a,b^	4.09 (2.70, 5.49) ^c,d^	0.12 (−0.93, 1.16) ^a,c^	−1.11 (−1.77, −0.45) ^b,d^
Visceral adiposity index	2.82 (2.25, 3.38) ^a^	3.65 (2.75, 4.56) ^b,c^	1.95 (1.46, 2.43) ^b^	1.51 (1.20, 1.82) ^a,c^

Data are presented as mean (95% confidence interval). HbA1c = glycated hemoglobin; HOMA-IR = homeostatic model assessment of insulin resistance; LDL-cholesterol = low-density lipoprotein cholesterol; HDL-cholesterol = high-density lipoprotein cholesterol. *p*-value was retrieved from a two-sided ANCOVA, adjusting for age and gender and with a post-hoc analysis of Tukey or Bonferroni depending on outcome normality. ^a, b, c, d^: Similar letters represent significant differences between groups (*p * <  0.05).

## Data Availability

Raw data will be uploaded upon www.zenodo.org immediately after the acceptance of the manuscript and they will be available upon reasonable request to the authors S.L. and A.S.

## Abbreviations

The following abbreviations are used in this manuscript:

BM	Body mass
BMI	Body mass index
BMR	Basal Metabolic Rate
BIA	Bioelectrical impedance analyser
BP	blood pressure
CRP	C-reactive protein
DBP	Diastolic blood pressure
EOB	Essential Obesity
FPG	Fasting plasma glucose
FFM	Fat free mass
FM	Fat mass
GH	Growth hormone
HDL-C	High-density lipoprotein cholesterol
Hb1Ac	Glycated hemoglobin
HOMA-IR	homeostatic model assessment of insulin resistance
LDL-C	Low-density lipoprotein cholesterol
MetS	Metabolic Syndrome
PWS	Prader Willi Syndrome
SBP	Sistolic blood pressure
TG	Triglycerides
WC	Waist circumference
VAI	Visceral Adiposity Index
